# Beyond the average patient: how neuroimaging models can address heterogeneity in dementia

**DOI:** 10.1093/brain/awab165

**Published:** 2021-04-23

**Authors:** Serena Verdi, Andre F Marquand, Jonathan M Schott, James H Cole

**Affiliations:** 1 Centre for Medical Image Computing, Medical Physics and Biomedical Engineering, University College London, London WC1V 6LJ, UK; 2 Dementia Research Centre, UCL Queen Square Institute of Neurology, London WC1N 3BG, UK; 3 Donders Centre for Cognitive Neuroimaging, Donders Institute for Brain, Cognition and Behaviour, Radboud University, Nijmegen, 6525EN, The Netherlands; 4 Department of Cognitive Neuroscience, Radboud University Medical Centre, Nijmegen, 6525EN, The Netherlands

**Keywords:** normative modelling, heterogeneity, precision medicine, clustering, dementia

## Abstract

Dementia is a highly heterogeneous condition, with pronounced individual differences in age of onset, clinical presentation, progression rates and neuropathological hallmarks, even within a specific diagnostic group. However, the most common statistical designs used in dementia research studies and clinical trials overlook this heterogeneity, instead relying on comparisons of group average differences (e.g. patient versus control or treatment versus placebo), implicitly assuming within-group homogeneity. This one-size-fits-all approach potentially limits our understanding of dementia aetiology, hindering the identification of effective treatments. Neuroimaging has enabled the characterization of the average neuroanatomical substrates of dementias; however, the increasing availability of large open neuroimaging datasets provides the opportunity to examine patterns of neuroanatomical variability in individual patients. In this update, we outline the causes and consequences of heterogeneity in dementia and discuss recent research that aims to tackle heterogeneity directly, rather than assuming that dementia affects everyone in the same way. We introduce spatial normative modelling as an emerging data-driven technique, which can be applied to dementia data to model neuroanatomical variation, capturing individualized neurobiological ‘fingerprints’. Such methods have the potential to detect clinically relevant subtypes, track an individual’s disease progression or evaluate treatment responses, with the goal of moving towards precision medicine for dementia.

## Teaser

Verdi *et al.* highlight heterogeneity in dementia and how it can cause problems for statistical design. An emerging alternative approach is introduced, namely spatial normative modelling, that uses neuroimaging to map individual differences to uncover heterogeneous patterns of brain changes in dementia patients.

## Introduction

**Figure 1:**
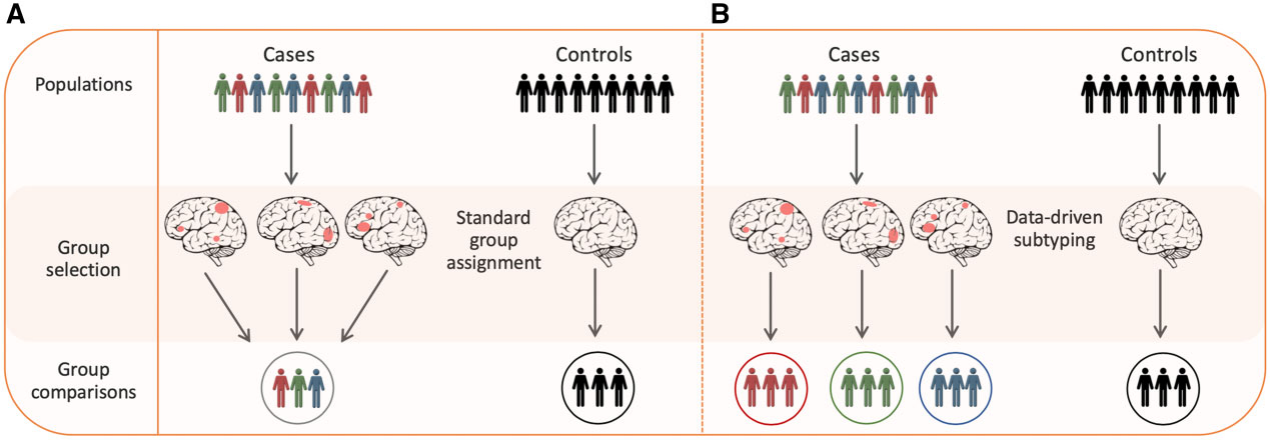
**Differences between case-control and data-driven subtype approaches.** (**A**) The conventional case-control approach. Despite underlying neurobiological heterogeneity, as illustrated by the red, green and blue (RGB) profiles, all patients are analysed together to calculate the group average. This is used to compare with healthy controls to highlight differences between the two groups. Here, the average patient (an average of RGB profiles, circled) assumes neurobiological homogeneity, potentially masking underlying subtypes or individual differences. (**B**) Data-driven neuroimaging approach. The cases present neurobiological heterogeneity and are subtyped according to their different neurobiological patterns. This informs the division of the case population into its respective subtypes (distinguished RGB profiles). These subtypes can then inform stratification for further investigation, such as clinical interventions, longitudinal monitoring or genome-wide association studies.

Heterogeneity is an underlying characteristic of the presentation and progression of dementia. Variability is observed in the underlying neuropathology, genetic risk factors, imaging and fluid biomarkers and in clinical and behavioural manifestations, reinforcing the idea that each dementia patient is unique. However, it is challenging to capture this heterogeneity when studying dementia and it is often not attempted. The conventional analytical approach focuses on characterizing group averages, not individual differences, assuming homogeneity between dementia patients (Fig. 1A). The failure to incorporate heterogeneity in statistical models of dementia may have limited our understanding of the pathophysiological mechanisms and slowed the development of treatments.

Despite thousands of treatment trials, only one drug (aducanumab) shows any promise for disease modification.^1^ This paucity of treatments, in combination with the rapid ageing of the global population, adds to the societal burden of dementia.^2^ This motivates the re-evaluation of common experimental approaches in dementia research and clinical trials with the goal of optimizing statistical design. In this update, we review current and emerging neuroimaging analysis methods that are able to account for the intrinsic heterogeneity, to help further our understanding of dementia and improve the prospect of developing effective treatments.

### Heterogeneity in dementia

Dementia is characterized by progressive cognitive decline, over and above that seen in normal ageing, with subsequent impact on activities of daily living. Dementia is the end point of multiple diseases, including Alzheimer’s disease, vascular dementia, Parkinson’s disease dementia, dementia with Lewy bodies, frontotemporal dementia and limbic-predominant age-related TDP-43 encephalopathy (LATE).^3^^,^^4^ Neuropathological factors vary between these diseases, though commonly include amyloid, tau and Lewy body accumulation^5^^,^^6^ and neurodegeneration.

The neuropathological hallmarks of the dementia syndromes have been well characterized in some cases, while others continue to be defined. Indeed, the Braak stages are an established method for characterizing and defining Alzheimer’s disease and Parkinson’s disease dementia pathology.^7^^,^^8^ However, neurobiological evidence suggests that there are many individual exceptions to these general rules. This could be because of the complex relationship between the clinical syndrome and underlying neuropathology, as well as individual differences in brain structure and function that predate the pathological onset. For instance, neocortical neuritic amyloid plaques have been observed post-mortem in over 50% of non-demented older adults, while almost 20% of dementia patients had no such plaques at death.^9^ Beyond amyloid, other dementia risk factors such as tau tangles, white matter lesions and vascular pathologies have been recorded in dementia-free older adults.^9^ This suggests that these pathological hallmarks of dementia are not universal, with some individuals resilient to insults that may be sufficient to cause dementia in others.

The mechanisms driving these pathological changes are yet to be fully determined, although they likely vary between individuals, both between and within diagnostic categories. Genetic and environmental risk factors also show considerable variability here.^10^^,^^11^ For example, *APOE* is the best-known genetic risk for sporadic Alzheimer’s disease but is only semi-dominant and moderately penetrant; at an age of 85 years, between 30% and 50% of *APOE* ε4 homozygotes do not have dementia.^12^ Potentially, different genetic and molecular mechanisms (or combinations of mechanisms) can result in dementia.

In addition, separate pathologies have broad phenotypic correspondence, and clinicopathological relationships can be varied (e.g. aphasia or behavioural disturbances in frontotemporal lobar degeneration).^13^ A specific dementia phenotype may be the result of different pathological processes, and conversely a single molecular pathology may result in multiple different dementia phenotypes.^14^ A specific diagnosis (e.g. Alzheimer’s disease) can also include pathological features characteristic of another dementia disease (e.g. TDP‐43 proteinopathy).^15^ Symptoms often do not conform to diagnostic boundaries.^16^^,^^17^ For instance, diverse symptoms are observed in frontotemporal lobar degeneration syndromes, but these do not easily fall within existing clinical categories or a single disease entity.^18^ Furthermore, heterogeneity can be seen in the severity of symptoms,^19^ rates of change^20^ and influence on activities of daily life.^21^

To add further complexity, many dementia patients have non-dementia comorbidities, for example, neuropsychiatric and gastrointestinal diagnoses,^22^ all of which may impact clinical presentation and disease progression. Age is also a key risk factor for pathophysiological changes; disentangling disease-related variation from the ageing process is challenging, for example when differentiating between normal cognitive decline or mild cognitive impairment as a prodromal phase of Alzheimer’s disease.^23^

Neuroimaging can offer *in vivo* neurobiological insights into the inter-individual variability in dementia.^24-26^ Structural MRI has uncovered anatomical differences in dementia patients, reflected in patterns of atrophy,^27^ and anatomical symmetry.^28^ Differences in pathological hallmarks can be seen using PET ligands; for instance, tau (e.g. ^18^F-AV1451) and amyloid (e.g. ^11^C-PiB) ligand binding has been shown to vary between patients with Alzheimer’s disease.^29^ Functional MRI has also been used to capture differences in connectivity.^30^

Neuroimaging is now commonly implemented in clinical trials to provide secondary outcome measures of treatment effectiveness.^31–33^ Here, the relationship between variance and statistical power is considered; variance can increase noise in the typical case-control designs and subsequently reduce statistical power to detect change. For example, in early trials of solanezumab, both amyloid-negative and amyloid-positive Alzheimer’s disease patients (defined using PET) were recruited, reducing the ability to detect the effects with high levels of sensitivity, as some patients were likely following different pathological trajectories.^34^^,^^35^ Subsequently, it has been argued that a stringent approach of selecting optimal participants and biomarkers of interest for clinical trials will increase the likelihood of success when evaluating average differences between patients.^36^ However, there are limitations to this approach. For example, hippocampal involvement in Parkinson’s disease dementia is widely disputed,^37^ and in Alzheimer’s disease both higher,^38^ or lower,^39^ caudate nucleus volumes have been reported compared with healthy controls. Such inconsistencies could be due to differences within patient groups in either the disease subtype or disease stage. Until both disease heterogeneity and disease dynamics are better understood, using biomarkers for stratified trial enrolment will likely remain contentious.

A key limitation of current statistical approaches is the assumption in traditional case-control studies that experimental groups are homogeneous, discrete entities (Fig. 1A). Here, tests of statistical significance are based on group means, generally regarding individual differences as error or noise. In other words, this approach is fundamentally oriented to comparing the ‘average patient’. Even in sophisticated multivariate analyses (e.g. machine learning), the focus tends to be on the discovery of canonical patterns across sets of variables that differentiate one group from another. This assumption of within-group homogeneity is neither reflected in real-world clinical populations nor in the heterogeneous pathological nature of neurodegenerative diseases. Inconsistencies are commonly seen in treatment effects^2^^,^^40^^,^^41^; however, this could be related to unmeasured individual differences rather than poor efficacy. The assumption that there are uniform effects of dementia or of treatment on the brain may be hindering the discovery of disease-modifying treatments, especially when translating to heterogeneous clinical settings. This motivates the incorporation of heterogeneity into trial designs.^42^ Given the importance of the brain in dementia, we outline ways to model heterogeneity using neuroimaging and illustrate the impact this could have on fundamental research and clinical trials.

### Data-driven statistical methods

Measuring and statistically modelling neurobiological heterogeneity in a clinical population requires large datasets. Fortunately, large neuroimaging datasets are increasingly available for dementia; these include the Alzheimer’s Disease Neuroimaging Initiative (ADNI), Open Access Series Of Imaging Studies (OASIS) and the National Alzheimer’s Coordinating Center (NACC). These datasets allow for more flexible and powerful statistical testing.^43–45^ These data have supported the development and application of novel data-driven methods designs in dementia research (Box 1).

Box 1 Data-driven statistical methods

Data-driven techniques seek to investigate the relationships between the data variables without imposing *a priori* knowledge of these relationships. Some machine learning techniques can be considered to offer a data-driven approach, whereby a computer automatically learns (e.g. update model parameters) to optimize performance from experience (i.e. examples of labelled data). This process involves discovering and exploiting regularities in ‘training data’. There are many different problems that can be approached by employing machine learning methods, including anomaly detection, clustering, classification and regression. Broadly speaking, techniques can be summarized into three main groups: supervised, semi-supervised or unsupervised learning. For supervised-learning algorithms, a set of input variables are associated with labels prior to estimating the model. For example, regression analyses (e.g. predicting continuous symptom scores) and classification tasks (e.g. discriminating patients from healthy controls) are examples of supervised learning. Unsupervised learning models the underlying latent structure or distribution in the data to uncover meaningful patterns without supplying a label for each data-point.

Clustering is considered unsupervised learning, because the input variable is unlabelled. Clustering aims to identify subtypes, which can be conceptualized as a way to parse a single heterogeneous dataset into a number of more homogeneous subsets (Fig. 1B). Models can be derived based on distance, density, connectivity and distribution of the data to be clustered, though such metrics tend to be correlated. Clustering has been the predominant data-driven approach used to explore heterogeneity in dementia.^46^ Numerous methods have been implemented, differing in the input features used, the clustering algorithms and the validation approach. Common clustering algorithms include agglomerative, graph-based and forest-tree based. For example, in agglomerative clustering; the proximity of individual data-points (e.g. based on tissue volumes or cortical thickness) are calculated, then similar clusters are merged together to form larger clusters, after which the proximity of new clusters is calculated; these steps are then iterated until all the clusters are merged together to form a single cluster.^78^ When implementing these methods, it is important to consider the type of data used (e.g. dimensionality of the data), the choice of algorithm and distance function, the order of the model, the clustering subspace and whether clusters are mutually exclusive (hard clustering) or probabilistic (soft clustering). After implementation, it is also important to assess the number and validity of the clusters generated.^65^

Semi-supervised learning can also be a powerful approach, using clustering techniques alongside supervised classification or regression models to bolster sensitivity in cases of limited training data (e.g. rare diseases). This is where training data-points can either be labelled or unlabelled, with unlabelled data-points aiding the learning of a better classifier (or vice versa). This can sometimes address common confounding effects (e.g. age and sex), which result in clustering the disease effect as transformation from the normal control distribution to the patient distribution, as opposed to just the largest factor of data variability.^25^

Recently, data-driven methods have enabled the estimation of disease subtypes from neuroimaging data, a promising way to disentangle heterogeneity by grouping patients by distinctive neurobiological and cognitive characteristics^46^ and disease progression.^47^^,^^48^ For instance, hierarchical clustering algorithms have been utilized to understand variation in cortical thickness,^49^^,^^50^ grey matter^51^ and progressive neurodegeneration.^52^ Clustering techniques employed on large Alzheimer’s disease neuroimaging datasets have suggested that there are disease subtypes with distinct patterns of cortical thinning. Atrophy-based groupings have been defined as either medial-temporal, parietal or widespread (‘diffuse’).^49^^,^^53^ Interestingly, these subtypes have also been associated with patterns of both amyloid^50^ and tau deposition^54^ and with cognitive phenotypes.^55^^,^^56^ In frontotemporal dementia, distinct atrophy subtypes have been reported, corresponding with the temporal-dominant, temporofrontoparietal, frontal-dominant and frontotemporal areas.^51^^,^^57^ In dementia with Lewy bodies, distinct atrophy subtypes have also been reported: non-atrophic, parietotemporal atrophy and occipitofrontal atrophy, with corresponding distinctions in cognitive performance.^58^^,^^59^ Despite some consistency between different reports, other studies have generated a different number of subtypes, while still others have reported subtypes with anatomical overlap, such as occipital areas overlapping with parietal and mild atrophic patterns.^60^

Most studies using clustering methods have analysed cross-sectional single time-point data; however, given the heterogeneity in disease progression, mapping longitudinal trajectories is an important focus for dementia research. Young and colleagues recently combined disease progression modelling and clustering techniques to enable inference of subtype and disease stage.^52^ Here, three distinct spatiotemporal atrophy patterns were observed in Alzheimer’s disease, with atrophy starting in either the medial temporal lobe, frontotemporal areas or basal ganglia. In addition, four distinct spatiotemporal atrophy patterns were observed in frontotemporal dementia, corresponding with different genetic subtypes.^52^ Future efforts could continue to explore heterogeneity in disease progression, including the examination of presymptomatic and prodromal disease phases. Parsing this longitudinal heterogeneity should enable stratification of dementia patients into groups with differing disease progression rates, with treatments and interventions tailored to these groups accordingly.

Defining biologically meaningful subtypes may have implications for fundamental research. Case-control genome-wide association studies are hindered by heterogeneity in patients and controls alike,^61^ and dementia is likely to be no exception. Restricting genome-wide association studies to more homogeneous subtypes should increase the sensitivity and reliability of such research to detect genetic risk factors for dementia.

Despite promising initial results from subtyping studies, there are key issues to consider prior to translating such models into clinical settings. The number of subtypes generated, subtype distinguishability and the stability of subtypes over the disease course should be considered.^46^ It is possible that subtypes may be confounded by statistical decisions (e.g. hyperparameter choices), technical factors (e.g. scanner), biomedical factors (e.g. age, sex or comorbid disease) or sampling bias. The validation of clustering-derived subtypes is challenging in the absence of ground truth.^62^ Therefore, to ensure that subtypes are biologically meaningful, external validation steps using independent datasets and long-term clinical outcome data (i.e. mortality rates and post-mortem data^63^) are essential. Recently, promising results have emerged using post-mortem histopathological data, which have yielded transdiagnostic disease clusters.^64^ However, the availability of datasets that enable such validation are limited, causing a bottleneck in the progress of dementia subtyping research. Hopefully, efforts to access existing hospital and community data will be successful in providing the data necessary for clinical validation.

It is also important to consider if discrete subtypes can explain the range of variability observed. By design, clustering assumes homogeneity within each cluster, which itself may not be valid.^65^ This motivates research, such as Zhang and colleagues’ Bayesian latent factor analysis, into less discrete or overlapping subtypes, with multiple subtype factors contributing to the explanation for the patterns of brain structure in any particular individual.^66^

### Spatial normative modelling

**Figure 2:**
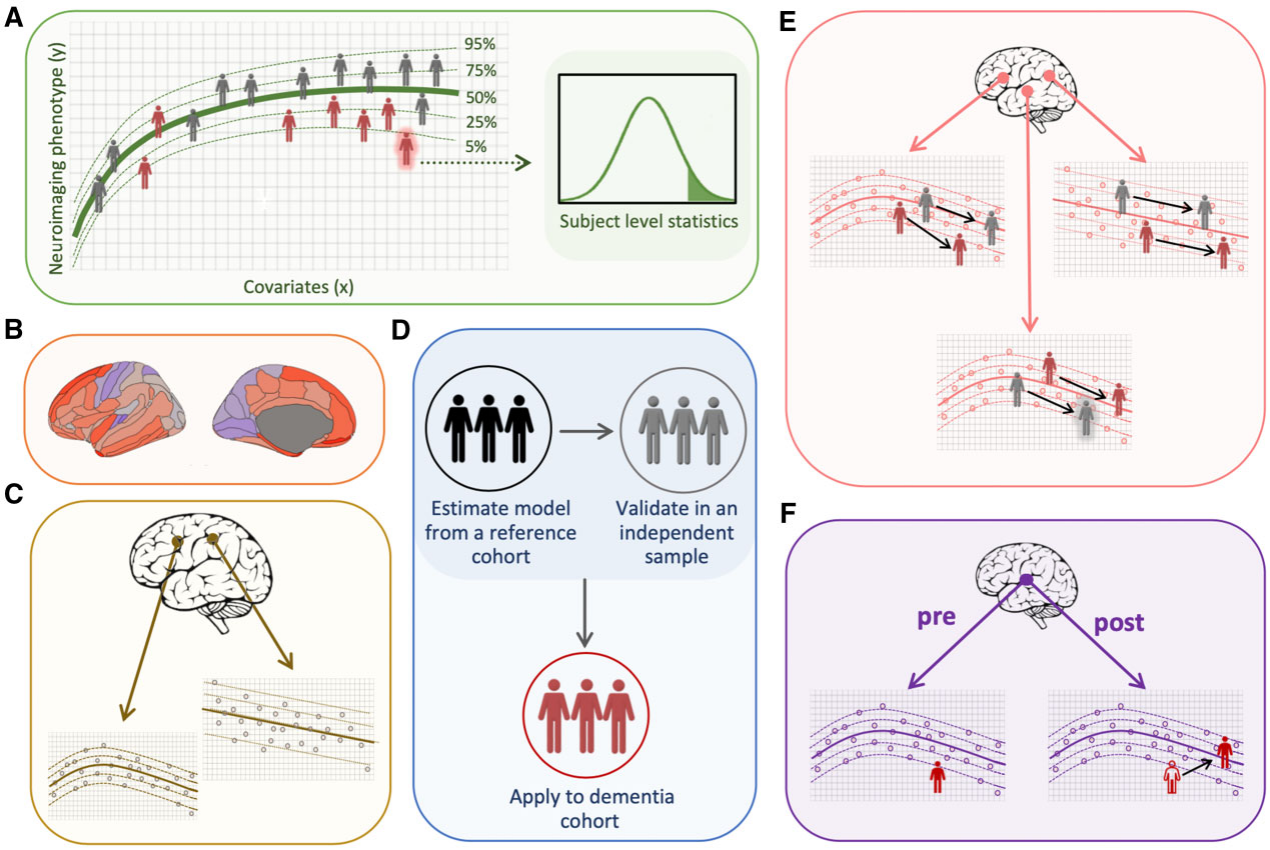
**Overview of spatial normative modelling.** (**A**) The spatial normative model maps centiles of variation across dementia patients and healthy controls. The Gaussian distribution curve (*right*) illustrates the statistical inference at the level of a dementia patient (red highlighted subject) with respect to the normative model. (**B**) An example individual *z*-score map for the left hemisphere based on cortical thickness. Red indicates thinner cortices, relative to the norm, and blue indicates thicker cortices. (**C**) Spatial inferences. Spatial normative models are estimated for each sampled brain location in regional space. This can be understood as a set of functions *y* = *f*(*x*), which uses covariates (*x*) to predict the regional neurobiological variable (*y*), derived from neuroimaging. (**D**) Procedural summary of spatial normative modelling. The spatial normative model is estimated using a healthy reference cohort. Next this is validated on withheld data (e.g. using cross-validation techniques), to ensure the accuracy of the model. The model then can be applied to a dementia cohort. (**E**) Detecting individual trajectories relative to the norm in three different brain regions. Longitudinal data can be used to observe how spatial differences change over time. (**F**) An example of a single spatial trajectory before and after an intervention in a clinical trial. Red = dementia group; grey = healthy subjects. For the graphs in **C**, **E** and **F**, unlabelled axis *x* = covariates, *y* = neurobiological variable.

Going further, it is possible to assess the neurobiology of dementia at the level of the individual patient and provide still greater precision than subtyping. To that end, normative modelling techniques have been developed to parse spatial heterogeneity (i.e. individual level regional variation) in neuroimaging data. Principally, normative modelling involves calculating the normal distribution of a population, then assessing how much an individual deviates from that respective distribution. Spatial normative modelling is a technique that specifically uses neuroimaging data (e.g. cortical thickness) to estimate variation for a given brain region.^67^^,^^68^ This is detailed in Box 2 and illustrated in Fig. 2. The extent to which an individual deviates from the norm can be spatially mapped at regions across the brain, providing an idiosyncratic map of individual variability. These ‘*z*-score’ maps can further be summarized to provide a patient-level index of deviation potentially reflecting their general brain health.

Box 2 Spatial normative modelling

Normative models provide statistical inferences at the level of the individual with respect to an expected ‘normative’ distribution or trajectory over time. This framework is commonly used in growth charts to map developmental changes in body weight and height as a function of age. Deviations from a normal growth curve manifest as outliers from the normative range at each age point.^79^ Specifically, spatial normative modelling adopts this concept by modelling the relationship between neurobiological variables (e.g. neuroimaging features which are represented in ‘space’) and covariates (e.g. demographic variables such as age and sex) to map centiles of variation across a cohort (Fig. 2A). An individual can then be located within the normative distribution to establish to what extent they are an outlier in a given measure. By applying this approach to derive spatial normative models at local brain regions, a map can be generated of where and to what extent an individual’s brain differs from the norm (Fig. 2B). Furthermore, by modelling the covariance across the normative cohort at each brain region, confidence intervals can be derived for each point prediction, giving a measure of uncertainty that can be useful for clinical interpretation and subsequent decision-making (Fig. 2D and E).

In the context of dementia, the process used to generate these individualized spatial normative brain maps could be as follows: Using a separate large reference dataset of healthy participants, spatial normative models of cortical thickness for separate brain regions can be statistically modelled based on age and sex. Next, the parameters of these models would be calibrated using cortical thickness measures derived from a subsample patient cohort under investigation (e.g. dementia patients and scanner-matched controls). From this, *z*-scores relative to the normative range would be generated for each brain region resulting in a brain ‘*z*-score map’ of cortical thickness for each participant in the remaining experimental sample (Fig. 2B and C).

These *z*-score maps could then be utilized in a variety of research or clinical settings. For example, patients could be ‘clustered’ based on these neuroanatomical patterns to provide biologically relevant subtypes that may have distinct clinical or biomarker signatures (Fig. 1B). This could provide a new mechanistic understanding of dementia as well as facilitate the discovery of genetic influences on dementia-related brain atrophy. Rather than assuming that dementia patients will show common patterns of brain changes, genome-wide association studies could attempt to identify genetic variants that distinguish these biologically more homogeneous subtypes from healthy controls. Such subtyping could also be used to stratify enrolment in clinical trials, including only specific subtypes to reduce heterogeneity and increase the power to detect average effects. This could substantially increase sensitivity to treatment effects, reducing the duration and costs of clinical trials.

Going beyond subtyping, the individual patient *z*-score maps could be used as surrogate outcome measures of treatment efficacy. Rather than simply assessing whether a treatment reduces hippocampal or whole-brain atrophy on average, the magnitude of longitudinal change in *z*-score maps could be compared between treatment and placebo groups. Importantly, this overcomes the assumption of homogeneity, i.e. that a treatment must affect all patients’ brains in the same way, slowing atrophy in the same regions. By capturing neuroanatomical heterogeneity at an individual level, spatial normative modelling could indicate whether a treatment slows brain atrophy in different regions in different people, whilst still generating standard effect sizes and confidence intervals for rigorous statistical evaluation.

Multiple algorithms have been proposed for spatial normative modelling. A common approach employs Gaussian process regression to estimate normative models in the brain.^67^ More recent developments have included a neural processes model, which does not rely on fixed parametric kernels and can improve the scaling of the model to large datasets. This method learns optimal feature representations and covariance structures for random-effect and noise (via global latent variables),^69^ and a hierarchical Bayesian regression approach to normative modelling has been shown to efficiently accommodate inter-site variation and provide computational scaling, which is useful when using large studies or combining smaller studies that are acquired across multiple sites.^70^

Preliminary studies have used normative modelling in the context of brain ageing and dementia. Distinct patterns of deviation from normal ranges were observed in people with mild cognitive impairment and patients with Alzheimer’s disease.^71^ Additionally, quantile regression techniques have been used to map deviations of cognitively normal individuals and brain morphology in patients with Alzheimer’s disease. Here, differences between these patients and healthy controls were partly attributed to accelerated ageing.^72^ Recently, a spatial pattern of atrophy index, reflecting normative variability, was used to demonstrate greater age-related atrophy in patients with Alzheimer’s disease compared with normative trends of age-related changes in brain structure.^73^

Spatial normative modelling has also been applied to neuroimaging data in the contexts of attention-deficit hyperactivity disorder,^74^ autism,^75^^,^^76^ bipolar disorder and schizophrenia.^77^ Results show that it is uncommon for patients to have uniform patterns of structural alterations across the brain. Individualized maps of regional differences derived from normative models generate distinct findings compared with case-control approaches, for example using voxel-based morphometry,^75^^,^^76^ which rely on modelling average differences between groups at each voxel. The predominant focus of neuroimaging research on group-level differences has potentially masked heterogeneity among patients within diagnostic groups.^74^^,^^77^ Therefore, spatial normative modelling provides a new approach to examining the neurobiological correlates of neurodevelopmental and psychiatric disorders and could well be applied in dementia.

As spatial normative modelling can utilize any continuous or categorical phenotype, a range of dementia-related neuroimaging features (i.e. local volumes, cortical thickness, diffusion microstructural indices or PET tracer binding) could be used in the model to ascertain the heterogeneity across multiple aspects of neurobiology. In addition, a multimodal approach can be adopted (e.g. combining variables of neuroanatomical volumetric measures and PET amyloid-β binding) to parse heterogeneity at both a molecular and structural level.

Going beyond neuroimaging, other data such as fluid biomarkers, physiological measures, cognitive assessments and genetic markers can be used to disentangle the heterogeneity in dementia. Such information could be incorporated explicitly in spatial normative models as predictors (alongside age and sex) of brain structure. For instance, tau CSF levels are currently pivotal in diagnosis and treatment planning; therefore, tau CSF could define a normative model of neuroanatomical measures to test whether elevated tau is associated with uniform or heterogeneous impacts on the brain. Alternatively, spatial normative models could be stratified, for example defining separate models for APOE4 carriers or people who are amyloid positive. While promising, these approaches require sufficiently large samples of normative data with that biomarker, which is potentially challenging when data collection involves invasive measures like CSF sampling. Importantly, non-imaging markers provide a means of validating subtypes from neuroimaging normative models, with the assumption that neuroanatomically homogeneous subtypes would be more homogeneous in terms of genetic and environmental risks and fluid biomarker readouts.

Furthermore, spatial normative modelling could be particularly informative with longitudinal data. Temporal heterogeneity could be modelled using two or more time points to understand within-subject changes. In this instance, greater changes in abnormality patterns could reflect faster disease progression (Fig. 2D). Similarly, evidence of reduced or reversed changes in abnormality patterns could be indicative of treatment efficacy, using an outcome measure tailored to each individual’s brain (Fig. 2E).

### Summary

In conclusion, while dementia is associated with marked clinical, aetiological and neuropathological variability, research studies and clinical trials often overlook this inherent heterogeneity. While neuroimaging has provided many insights into the neuroanatomy of dementia and has helped to assess treatment efficacy, the reliance on group-average statistical methods may have hindered efforts to understand the aetiology and prognosis, which have led to failures in clinical drug development. We have outlined how data-driven neuroimaging statistical techniques enable explicit modelling of heterogeneity in the brain. We propose that the application of spatial normative modelling methods to dementia neuroimaging studies is a promising avenue to mapping regional variations at the individual level; efforts should include an investigation of various neuroanatomical markers derived from neuroimaging, which are then validated using independent datasets. Here, our clinicopathological understanding of anatomical variation could be enhanced by multimodal neuroimaging techniques and combining other biological data as predictors in normative models. Importantly, these spatial normative modelling techniques are not designed to replace or even improve on diagnoses based on clinical evidence and well-established biomarkers. The goal here is to better capture the variability within diagnostic groups based on individual patterns of brain structure or potentially define neuroanatomical subtypes rather than span diagnostic boundaries. Employing these methods could be highly advantageous in mapping neurobiological abnormalities. In particular, the use of serial neuroimaging to define patient level longitudinal trajectories of neuroanatomical variability has the potential to improve predictions of disease progression or treatment response at the level of the individual patient, thereby paving the way towards more effective, precise medicine for dementia.

## Funding

This work was supported by the EPSRC-funded UCL Centre for Doctoral Training in Intelligent, Integrated Imaging in Healthcare (i4health) (EP/S021930/1) and the Department of Health’s National Institute for Health Research funded University College London Hospitals Biomedical Research Centre. In addition, A.F.M. gratefully acknowledges funding from the Dutch Organization for Scientific Research via a VIDI fellowship (grant number 016.156.415); J.M.S. acknowledges the support of Alzheimers Research UK, Brain Research UK, Weston Brain Institute, Medical Research Council and the British Heart Foundation.

## Competing interests

The authors report no competing interests.

## References

[1] Schneider L. A resurrection of aducanumab for Alzheimer’s disease. Lancet Neurol. 2020;19(2):111–112. 31978357 10.1016/S1474-4422(19)30480-6

[2] Gauthier S , Albert M, Fox N, et al Why has therapy development for dementia failed in the last two decades? Alzheimers Dement. 2016;12(1):60–64. 26710325 10.1016/j.jalz.2015.12.003

[3] Burns A , Iliffe S. Alzheimer’s disease. BMJ. 2009;338(1):b158. 19196745 10.1136/bmj.b158

[4] Nelson PT , Dickson DW, Trojanowski JQ, et al Limbic-predominant age-related TDP-43 encephalopathy (LATE): Consensus working group report. Brain. 2019;142(6):1503–1527. 31039256 10.1093/brain/awz099 PMC6536849

[5] Weller J , Budson A. Current understanding of Alzheimer’s disease diagnosis and treatment. F1000Res. 2018;7:F1000 Faculty Rev-1161.

[6] Armstrong MJ. Lewy body dementias. Continuum (Minneap Minn). 2019;25(1):128–146. 30707190 10.1212/CON.0000000000000685

[7] Braak H , Braak E. Neuropathological stageing of Alzheimer-related changes. Acta Neuropathologica. 1991;82(4):239–259. 1759558 10.1007/BF00308809

[8] Braak H , Del Tredici K, Rüb U, De Vos RAI, Jansen Steur ENH, Braak E. Staging of brain pathology related to sporadic Parkinson’s disease. Neurobiol Aging. 2003;24(2):197–211. 12498954 10.1016/s0197-4580(02)00065-9

[9] Savva GM , Wharton SB, Ince PG, Forster G, Matthews FE, Brayne C. Age, neuropathology, and dementia. New Engl J Med. 2009;360(22):2302–2309. 19474427 10.1056/NEJMoa0806142

[10] Cohen-Mansfield J. Heterogeneity in dementia: Challenges and opportunities. Alzheimer Dis Assoc Disorders. 2000;14(2):60–63. 10.1097/00002093-200004000-00002 10850743

[11] Ringman JM , Goate A, Masters CL, et al; Dominantly Inherited Alzheimer Network. Genetic heterogeneity in Alzheimer disease and implications for treatment strategies. Curr Neurol Neurosci Rep. 2014;14(11):499. 25217249 10.1007/s11910-014-0499-8 PMC4162987

[12] Genin E , Hannequin D, Wallon D, et al APOE and Alzheimer disease: A major gene with semi-dominant inheritance. Mol Psychiatry. 2011;16(9):903–907. 21556001 10.1038/mp.2011.52 PMC3162068

[13] Lam B , Masellis M, Freedman M, Stuss DT, Black SE. Clinical, imaging, and pathological heterogeneity of the Alzheimer’s disease syndrome. Alzheimers Res Ther. 2013;5(1):1. 23302773 10.1186/alzrt155 PMC3580331

[14] Rabinovici GD , Carrillo MC, Forman M, et al Multiple comorbid neuropathologies in the setting of Alzheimer’s disease neuropathology and implications for drug development. Alzheimers Dement. 2017;3(1):83–91. 10.1016/j.trci.2016.09.002 PMC5651346 29067320

[15] Thomas DX , Bajaj S, McRae-McKee K, Hadjichrysanthou C, Anderson RM, Collinge J. Association of TDP-43 proteinopathy, cerebral amyloid angiopathy, and Lewy bodies with cognitive impairment in individuals with or without Alzheimer’s disease neuropathology. Sci Rep. 2020;10(1):14579. 10.1038/s41598-020-71305-2 PMC7471113 32883971

[16] Cunningham EL , McGuinness B, Herron B, Passmore AP. Dementia . Ulster Med J. 2015;84(2):79–87. 26170481 PMC4488926

[17] Piguet O. Neither white nor black: Embracing clinical variability in dementia diagnosis. Brain. 2020;143(5):1291–1293. 32438412 10.1093/brain/awaa119

[18] Murley AG , Coyle-Gilchrist I, Rouse MA, et al Redefining the multidimensional clinical phenotypes of frontotemporal lobar degeneration syndromes. Brain. 2020;143(5):1555–1571. 32438414 10.1093/brain/awaa097 PMC7241953

[19] Ryan J , Fransquet P, Wrigglesworth J, Lacaze P. Phenotypic heterogeneity in dementia: A challenge for epidemiology and biomarker studies. Front Public Health. 2018;6:181. 29971228 10.3389/fpubh.2018.00181 PMC6018385

[20] Goyal D , Tjandra D, Migrino RQ, Giordani B, Syed Z, Wiens J.; Alzheimer's Disease Neuroimaging Initiative. Characterizing heterogeneity in the progression of Alzheimer’s disease using longitudinal clinical and neuroimaging biomarkers. Alzheimers Dement. 2018;10(1):629–637. 10.1016/j.dadm.2018.06.007 PMC6234900 30456290

[21] Urwyler P , Stucki R, Rampa L, Müri R, Mosimann UP, Nef T. Cognitive impairment categorized in community-dwelling older adults with and without dementia using in-home sensors that recognise activities of daily living. Sci Rep. 2017;7:42084. 28176828 10.1038/srep42084 PMC5296716

[22] Bunn F , Burn AM, Goodman C, et al Comorbidity and dementia: A scoping review of the literature. BMC Med. 2014;12(1):192. 25358236 10.1186/s12916-014-0192-4 PMC4229610

[23] Toepper M. Dissociating normal aging from Alzheimer’s disease: A view from cognitive neuroscience. J Alzheimers Dis. 2017;57(2):331–352. 28269778 10.3233/JAD-161099 PMC5366251

[24] Cash DM , Rohrer JD, Ryan NS, Ourselin S, Fox NC. Imaging endpoints for clinical trials in Alzheimer’s disease. Alzheimers Res Ther. 2014;6(9). 10.1186/s13195-014-0087-9 PMC4304258 25621018

[25] Dong A , Toledo JB, Honnorat N, et al; Alzheimer’s Disease Neuroimaging Initiative. Heterogeneity of neuroanatomical patterns in prodromal Alzheimer’s disease: Links to cognition, progression and biomarkers. Brain. 2017;140(3):735–747. 28003242 10.1093/brain/aww319 PMC5837514

[26] Weiner MW , Veitch DP, Aisen PS, et al; Alzheimer's Disease Neuroimaging Initiative. Recent publications from the Alzheimer’s Disease Neuroimaging Initiative: Reviewing progress toward improved AD clinical trials. Alzheimers Dement. 2017;13(4):e1–e85. 28342697 10.1016/j.jalz.2016.11.007 PMC6818723

[27] Whitwell JL , Dickson DW, Murray ME, et al Neuroimaging correlates of pathologically defined subtypes of Alzheimer’s disease: A case-control study. Lancet Neurol. 2012;11(10):868–877. 22951070 10.1016/S1474-4422(12)70200-4 PMC3490201

[28] Mesulam MM , Weintraub S, Rogalski EJ, Wieneke C, Geula C, Bigio EH. Asymmetry and heterogeneity of Alzheimer’s and frontotemporal pathology in primary progressive aphasia. Brain. 2014;137(Pt 4):1176–1192. 24574501 10.1093/brain/awu024 PMC3959558

[29] Ossenkoppele R , Schonhaut DR, Schöll M, et al Tau PET patterns mirror clinical and neuroanatomical variability in Alzheimer’s disease. Brain. 2016;139(5):1551–1567. 26962052 10.1093/brain/aww027 PMC5006248

[30] Khan W , Amad A, Giampietro V, et al; Alzheimer's Disease Neuroimaging Initiative. The heterogeneous functional architecture of the posteromedial cortex is associated with selective functional connectivity differences in Alzheimer’s disease. Hum Brain Mapp. 2020;41(6):1557–1572. 31854490 10.1002/hbm.24894 PMC7268042

[31] Klunk WE , Engler H, Nordberg A, et al Imaging brain amyloid in Alzheimer’s disease with Pittsburgh compound-B. Ann Neurol. 2004;55(3):306–319. 14991808 10.1002/ana.20009

[32] Valenzuela M , Bartrés-Faz D, Beg F, et al Neuroimaging as endpoints in clinical trials: Are we there yet? Perspective from the first Provence workshop. Mol Psychiatry. 2011;16(11):1064–1066. 21625230 10.1038/mp.2011.62

[33] Staffaroni AM , Ljubenkov PA, Kornak J, et al Longitudinal multimodal imaging and clinical endpoints for frontotemporal dementia clinical trials. Brain. 2019;142(2):443–459. 30698757 10.1093/brain/awy319 PMC6351779

[34] Siemers ER , Sundell KL, Carlson C, et al Phase 3 solanezumab trials: Secondary outcomes in mild Alzheimer’s disease patients. Alzheimers Dement. 2016;12(2):110–120. 26238576 10.1016/j.jalz.2015.06.1893

[35] Honig LS , Vellas B, Woodward M, et al Trial of solanezumab for mild dementia due to Alzheimer’s disease. New Engl J Med. 2018;378(4):321–330. 29365294 10.1056/NEJMoa1705971

[36] Cummings J , Feldman HH, Scheltens P. The "rights" of precision drug development for Alzheimer’s disease. Alzheimers Res Ther. 2019;11(1):1–14. 31470905 10.1186/s13195-019-0529-5 PMC6717388

[37] Weil RS , Hsu JK, Darby RR, Soussand L, Fox MD. Neuroimaging in Parkinson’s disease dementia: Connecting the dots. Brain Commun. 2019;1(1):fcz006. 10.1093/braincomms/fcz006 PMC6777517 31608325

[38] Persson K , Selbæk G, Brækhus A, Beyer M, Barca M, Engedal K. Fully automated structural MRI of the brain in clinical dementia workup. Acta Radiol. 2017;58(6):740–747. 27687251 10.1177/0284185116669874

[39] Jiji S , Smitha KA, Gupta AK, Pillai VPM, Jayasree RS. Segmentation and volumetric analysis of the caudate nucleus in Alzheimer’s disease. Eur J Radiol. 2013;82(9):1525–1530. 23664648 10.1016/j.ejrad.2013.03.012

[40] Cummings J , Aisen PS, Dubois B, et al Drug development in Alzheimer’s disease: The path to 2025. Alzheimers Res Ther. 2016;8:1–12. 27646601 10.1186/s13195-016-0207-9 PMC5028936

[41] Cummings J , Lee G, Ritter A, Sabbagh M, Zhong K. Alzheimer’s disease drug development pipeline: 2019. Alzheimers Dement. 2019;5(1):272–293. 10.1016/j.trci.2019.05.008 PMC6617248 31334330

[42] Aisen PS , Cummings J, Jack CR, et al On the path to 2025: Understanding the Alzheimer’s disease continuum. Alzheimers Res Ther. 2017;9(1):60. 10.1186/s13195-017-0283-5 PMC5549378 28793924

[43] Weiner MW , Aisen PS, Jack CR, et al The Alzheimer’s disease neuroimaging initiative: Progress report and future plans. Alzheimers Dement. 2010;6(3):202–11.e7. 20451868 10.1016/j.jalz.2010.03.007 PMC2927112

[44] Marek K , Chowdhury S, Siderowf A, et al; The Parkinson's Progression Markers Initiative. The Parkinson’s progression markers initiative (PPMI) – establishing a PD biomarker cohort. Ann Clin Transl Neurol. 2018;5(12):1460–1477. 30564614 10.1002/acn3.644 PMC6292383

[45] LaMontagne P , Benzinger T, Morris J et al OASIS-3: Longitudinal Neuroimaging, Clinical, and Cognitive Dataset for Normal Aging and Alzheimer Disease. *medRxiv*. [Preprint] doi:10.1101/2019.12.13.19014902

[46] Habes M , Grothe MJ, Tunc B, McMillan C, Wolk DA, Davatzikos C. Disentangling heterogeneity in Alzheimer’s disease and related dementias using data-driven methods. Biol Psychiatry. 2020;88(1):70–82. 32201044 10.1016/j.biopsych.2020.01.016 PMC7305953

[47] Oxtoby NP , Young AL, Cash DM, et al Data-driven models of dominantly-inherited Alzheimer’s disease progression. Brain. 2018;141(5):1529–1544. 29579160 10.1093/brain/awy050 PMC5920320

[48] Oxtoby NP , Leyland L, Aksman LM, et al Sequence of clinical and neurodegeneration events in Parkinson’s disease progression. Brain. 2021;144(3):975–988. 33543247 10.1093/brain/awaa461 PMC8041043

[49] Noh Y , Jeon S, Lee JM, et al Anatomical heterogeneity of Alzheimer disease Based on cortical thickness on MRIs. Neurology. 2014;83(21):1936–1944. 25344382 10.1212/WNL.0000000000001003 PMC4248459

[50] Hwang J , Kim CM, Jeon S, et al; Alzheimer's Disease Neuroimaging Initiative. Prediction of Alzheimer’s disease pathophysiology based on cortical thickness patterns. Alzheimers Dement. 2016;2:58–67. 10.1016/j.dadm.2015.11.008 PMC4879518 27239533

[51] Whitwell JL , Przybelski SA, Weigand SD, et al Distinct anatomical subtypes of the behavioural variant of frontotemporal dementia: A cluster analysis study. Brain. 2009;132(11):2932–2946. 19762452 10.1093/brain/awp232 PMC2768663

[52] Young AL , Marinescu RV, Oxtoby NP, et al; The Genetic FTD Initiative (GENFI). Uncovering the heterogeneity and temporal complexity of neurodegenerative diseases with Subtype and Stage Inference. Nat Commun. 2018;9(1):10.1038/s41467-018-05892-0 PMC6189176 30323170

[53] Na HK , Kang DR, Kim S, et al Malignant progression in parietal-dominant atrophy subtype of Alzheimer’s disease occurs independent of onset age. Neurobiol Aging. 2016;47:149–156. 27592283 10.1016/j.neurobiolaging.2016.08.001

[54] Jeon S , Kang JM, Seo S, et al Topographical heterogeneity of Alzheimer’s disease based on MR imaging, tau PET, and amyloid PET. Front Aging Neurosci. 2019;11:211. 10.3389/fnagi.2019.00211 PMC6710378 31481888

[55] Park JY , Na HK, Kim S, et al; Alzheimer’s Disease Neuroimaging Initiative. Robust Identification of Alzheimer’s Disease subtypes based on cortical atrophy patterns. Sci Rep. 2017;7:43270. 28276464 10.1038/srep43270 PMC5343676

[56] Sun N , Mormino EC, Chen J, Sabuncu MR, Yeo BTT; Alzheimer’s Disease Neuroimaging Initiative. Multi-modal latent factor exploration of atrophy, cognitive and tau heterogeneity in Alzheimer’s disease. Neuroimage. 2019;201:116043. 31344486 10.1016/j.neuroimage.2019.116043

[57] Whitwell JL , Xu J, Mandrekar J, et al Frontal asymmetry in behavioral variant frontotemporal dementia: Clinicoimaging and pathogenetic correlates. Neurobiol Aging. 2013;34(2):636–639. 22502999 10.1016/j.neurobiolaging.2012.03.009 PMC3404265

[58] Uribe C , Segura B, Baggio HC, et al Patterns of cortical thinning in nondemented Parkinson’s disease patients. Mov Disord. 2016;31(5):699–708. 27094093 10.1002/mds.26590 PMC5061099

[59] Uribe C , Segura B, Baggio HC, et al Progression of Parkinson’s disease patients’ subtypes based on cortical thinning: 4-year follow-up. Parkinson Relat Disord. 2019;64:286–292. 10.1016/j.parkreldis.2019.05.012 31103485

[60] Ten Kate M , Dicks E, Visser PJ, et al; Alzheimer’s Disease Neuroimaging Initiative. Atrophy subtypes in prodromal Alzheimer’s disease are associated with cognitive decline. Brain. 2018;141(12):3443–3456. 30351346 10.1093/brain/awy264 PMC6669409

[61] Manchia M , Cullis J, Turecki G, Rouleau GA, Uher R, Alda M. The impact of phenotypic and genetic heterogeneity on results of genome wide association studies of complex diseases. PLoS One. 2013;8(10):e76295 24146854 10.1371/journal.pone.0076295 PMC3795757

[62] Badhwar AP , Peggy McFall G, Sapkota S, et al A multiomics approach to heterogeneity in Alzheimer’s disease: Focused review and roadmap. Brain. 2020;143(5):1315–1331. 31891371 10.1093/brain/awz384 PMC7241959

[63] Harper L , Fumagalli GG, Barkhof F, et al MRI visual rating scales in the diagnosis of dementia: Evaluation in 184 post-mortem confirmed cases. Brain. 2016;139(4):1211–1225. 26936938 10.1093/brain/aww005 PMC4806219

[64] Cornblath EJ , Robinson JL, Irwin DJ, et al Defining and predicting transdiagnostic categories of neurodegenerative disease. Nat Biomed Eng. 2020;4(8):787–800. 32747831 10.1038/s41551-020-0593-y PMC7946378

[65] Marquand AF , Wolfers T, Mennes M, Buitelaar J, Beckmann CF. Beyond lumping and splitting: A review of computational approaches for stratifying psychiatric disorders. Biol Psychiatry Cogn Neurosci Neuroimaging. 2016;1(5):433–447. 27642641 10.1016/j.bpsc.2016.04.002 PMC5013873

[66] Zhang X , Mormino EC, Sun N, Sperling RA, Sabuncu MR, Yeo BTT; Alzheimer’s Disease Neuroimaging Initiative. Bayesian model reveals latent atrophy factors with dissociable cognitive trajectories in Alzheimer’s disease. Proc Natl Acad Sci U S A. 2016;113(42):E6535–E6544. 27702899 10.1073/pnas.1611073113 PMC5081632

[67] Marquand AF , Rezek I, Buitelaar J, Beckmann CF. Understanding heterogeneity in clinical cohorts using normative models: Beyond case-control studies. Biol Psychiatry. 2016;80(7):552–561. 26927419 10.1016/j.biopsych.2015.12.023 PMC5023321

[68] Marquand AF , Kia SM, Zabihi M, Wolfers T, Buitelaar JK, Beckmann CF. Conceptualizing mental disorders as deviations from normative functioning. Mol Psychiatry. 2019;24(10):1415–1424. 31201374 10.1038/s41380-019-0441-1 PMC6756106

[69] Kia SM , Marquand AF. Neural processes mixed-effect models for deep normative modeling of clinical neuroimaging data. In: *Proceedings of The 2nd International Conference on Medical Imaging with Deep Learning*. PMLR. 2019;102:297-314.

[70] Kia SM , Huijsdens H, Dinga R et al Hierarchical Bayesian regression for multi-site normative modeling of neuroimaging data. *arXiv*. 2020. https://arxiv.org/abs/2005.12055

[71] Ziegler G , Ridgway GR, Dahnke R, Gaser C.; Alzheimer's Disease Neuroimaging Initiative. Individualized Gaussian process-based prediction and detection of local and global gray matter abnormalities in elderly subjects. Neuroimage. 2014;97:333–348. 24742919 10.1016/j.neuroimage.2014.04.018 PMC4077633

[72] Huizinga W , Poot DHJ, Vernooij MW, et al Alzheimer's Disease Neuroimaging Initiative. A spatio-temporal reference model of the aging brain. Neuroimage. 2018;169:11–22. 29203452 10.1016/j.neuroimage.2017.10.040

[73] Habes M , Pomponio R, Shou H et al The Brain Chart of Aging: Machine-learning analytics reveals links between brain aging, white matter disease, amyloid burden, and cognition in the iSTAGING consortium of 10,216 harmonized MR scans. *Alzheimer’s Dement*. 2021;17(1):89–102. 10.1002/alz.12178 PMC7923395 32920988

[74] Wolfers T , Beckmann CF, Hoogman M, Buitelaar JK, Franke B, Marquand AF. Individual differences v. the average patient: Mapping the heterogeneity in ADHD using normative models. Psychol Med. 2020;50(2):314–323. 30782224 10.1017/S0033291719000084 PMC7083555

[75] Zabihi M , Oldehinkel M, Wolfers T, et al Dissecting the heterogeneous cortical anatomy of autism spectrum disorder using normative models. Biol Psychiatry Cogn Neurosci Neuroimaging. 2019;4(6):567–578. 30799285 10.1016/j.bpsc.2018.11.013 PMC6551348

[76] Bethlehem RAI , Seidlitz J, Romero-Garcia R, Trakoshis S, Dumas G, Lombardo MV. A normative modelling approach reveals age-atypical cortical thickness in a subgroup of males with autism spectrum disorder. Commun Biol. 2020;3(1):486. 10.1038/s42003-020-01212-9 PMC7474067 32887930

[77] Wolfers T , Doan NT, Kaufmann T, et al Mapping the heterogeneous phenotype of schizophrenia and bipolar disorder using normative models. JAMA Psychiatry. 2018;75(11):1146–1155. 30304337 10.1001/jamapsychiatry.2018.2467 PMC6248110

[78] Murtagh F , Legendre P. Ward’s hierarchical agglomerative clustering method: Which algorithms implement ward’s criterion? J Classif. 2014;31(3):274–295.

[79] Cole TJ. The development of growth references and growth charts. Ann Hum Biol. 2012;39(5):382–394. 22780429 10.3109/03014460.2012.694475 PMC3920659

